# Surgical case report—acalculous hemorrhagic cholecystitis

**DOI:** 10.1093/jscr/rjab075

**Published:** 2021-03-25

**Authors:** Matthew Leaning

**Affiliations:** Department of General Surgery, Caboolture Hospital, Brisbane, Queensland, Australia

**Keywords:** acalculous, haemorrhagic, cholecystitis, cholecystectomy

## Abstract

Haemorrhagic cholecystitis is a seldom seen cause of right upper quadrant pain that can result in gallbladder rupture, massive intraperitoneal haemorrhage and death if untreated. Haemorrhagic cholecystitis is usually seen in the presence of cholelithiasis, malignancy, trauma and coagulopathies. Here, we present the unusual case of an elderly man presenting with acalculous haemorrhagic cholecystitis, who was successfully treated with laparoscopic cholecystectomy. We review the radiological and laparoscopic findings of haemorrhagic acalculous cholecystitis. This case highlights the importance of prudent use of radiological imaging to differentiate haemorrhagic cholecystitis from alternate pathology and early surgical intervention to avoid massive intraperitoneal haemorrhage and the high mortality with which it is associated.

## INTRODUCTION

Haemorrhagic cholecystitis is an infrequent cause of right upper quadrant pain with a reported mortality rate of 15–20% [1]. An awareness of this rare condition is paramount, given the high mortality rate associated with it and its ability to mimic a variety of other pathologies. If missed, haemorrhagic cholecystitis can result in massive intraperitoneal haemorrhage and death [2]. The largest published series of cases found it was associated with anticoagulation in 45% of the cases [3]. Other reported risk factors include trauma, cirrhosis, vasculitides, malignancy and chronic renal impairment. Treatment is typically cholecystectomy with some evidence for cholecystostomy [3]. Here, we present an atypical case of acalculous haemorrhagic cholecystitis.

## CASE REPORT

A 73–year-old gentleman presented to the emergency department with 1 day of sudden-onset right upper quadrant pain, nausea and vomiting. His past medical history included pulmonary emboli, chronic obstructive pulmonary disease (COPD), ischaemic stroke, hypertension, chronic kidney disease and reflux. He was anticoagulated on apixaban, was a current heavy smoker and had a history of alcohol excess. Other medications included omeprazole, metoprolol and prazosin.

On examination, his abdomen was soft with right upper quadrant tenderness, a negative Murphy’s sign and no peritonism. Observations demonstrated he was afebrile, hypertensive and normocardic. Admission blood tests revealed a normal haemoglobin, inflammatory markers, liver function tests and lipase with a chronic, unchanged renal impairment; estimated glomerular filtration rate (eGFR): 35 ml/min.

**Figure 1:**
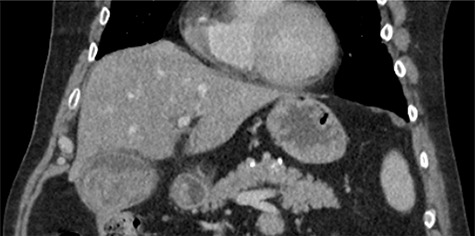
Coronal non-contrast CT of abdomen on day of presentation, showing hyperdense nodular soft tissue filling the gallbladder lumen.

The emergency physicians arranged an urgent non-contrast computerized tomography (CT) scan of the abdomen and pelvis. This revealed hyperdense nodular soft tissue filling the gallbladder lumen with a non-thickened but prominent gallbladder wall, as shown in Fig. 1. There was biliary dilatation, with extensive fat stranding and fluid surrounding the gallbladder neck, porta hepatic region and common bile duct. The reporting radiologist’s differentials included malignancy or haemorrhagic cholecystitis, and magnetic resonance imaging (MRI) was advised.

**Figure 2:**
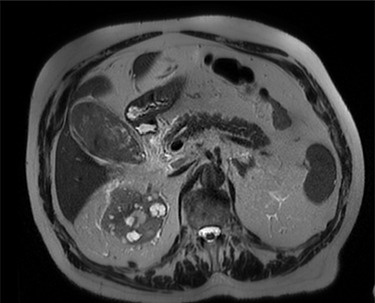
MRI scan of the patient, showing a distended gallbladder with low T1 and T2 content demonstrating no internal enhancement.

**Figure 3:**
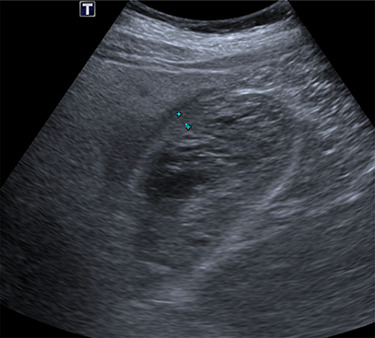
US scan of the patient, demonstrating a thick-walled gallbladder, with no evidence of calculi.

Intravenous broad-spectrum antibiotics were commenced, and the patient was admitted to the surgical ward and fasted for an MRI liver with gadolinium contrast. The MRI revealed no convincing features of malignancy; however, the gallbladder was distended with heterogeneous, predominantly low T1 and T2 content and demonstrating no internal enhancement, as seen in Fig. 2. These findings suggested the presence of sludge and/or luminal blood clod, and a further ultrasound (US) of the gallbladder was recommended. US scan, as shown in Fig. 3, confirmed cholecystitis with no evidence of cholelithiasis.

**Figure 4:**
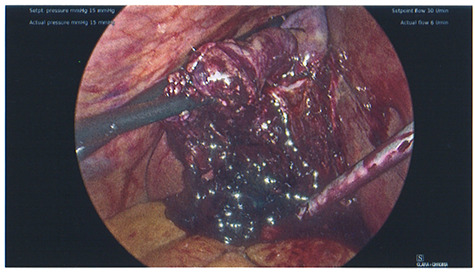
Photo taken intraoperatively, demonstrating haematoma leaking from a necrotic gallbladder.

The patient was taken to theatre the second day of the admission. At laparoscopy, the gallbladder was visibly necrotic. The gallbladder was inadvertently perforated during retraction and a large volume haematoma was evacuated, as shown in Fig. 4. Dissection proceeded normally and the critical view was achieved. No intraoperative cholangiogram was performed due to the inflamed and friable nature of Calot’s triangle. The cystic duct and artery were clipped and cut, allowing the gallbladder to be dissected off the cystic plate. Surgicell was placed in the gallbladder fossa and a 15fr drain was secured. The drain was subsequently removed the day following operation, and on Day 2, the patient was discharged on a normal diet, having restarted apixaban.

Histological examination confirmed extensive serosal haemorrhage, acute gangrenous cholecystitis with focal full thickness necrosis and no cholelithiasis.

## DISCUSSION

Documented risk factors for the development of haemorrhagic cholecystitis include anticoagulation, a history of trauma, malignancy, coagulopathies, cirrhosis and renal failure [4]. In this case, the patient was anticoagulated. Rather atypically, this patient had no evidence of co-existing cholelithiasis intraoperatively or histologically. The author found only one other reported case of acalculous, atraumatic haemorrhagic cholecystitis in the literature, which was reported in Korean [5].

Haemorrhagic cholecystitis produces a range of presentations, typically right upper quadrant pain, fevers and raised inflammatory markers [6]. The pathophysiology is not well understood but is presumed to proceed similarly to other forms of acute cholecystitis [7, 8]. Haemorrhage into the gallbladder lumen arises typically from an inflammatory process of the gallbladder wall or trauma, resulting in damage to the mucosal blood vessels and haematoma formation [7]. This bleeding together with the obstruction of the cystic duct results in distension, and thus ischaemic necrosis of the gallbladder wall, and if untreated, gallbladder perforation and massive intraperitoneal haemorrhage [7]. Given there was no cholelithiasis present and no history of trauma, the cause of the intraluminal haematoma in this patient was not clear.

Multiple treatment modalities exist for the management of haemorrhagic cholecystitis. In the majority of reported cases, patients have undergone either open or laparoscopic cholecystectomy, and this approach is associated with superior outcomes [3, 4, 6–9]. In patients who are poor surgical candidates, radiological and endoscopic decompression of the gallbladder has been utilized successfully as well as medical management alone [3–10].

Acalculous haemorrhagic cholecystitis is a seldom seen and challenging condition. Diligent use of multiple imaging modalities and early recognition and intervention are paramount in preventing the most fearsome complications of haemorrhagic cholecystitis: massive intraperitoneal haemorrhage.

## CONFLICT OF INTEREST STATEMENT

None declared.

## FUNDING

None.
